# Choosing the Most Effective Pattern Classification Model under Learning-Time Constraint

**DOI:** 10.1371/journal.pone.0129947

**Published:** 2015-06-26

**Authors:** Priscila T. M. Saito, Rodrigo Y. M. Nakamura, Willian P. Amorim, João P. Papa, Pedro J. de Rezende, Alexandre X. Falcão

**Affiliations:** 1 Department of Computing, Federal University of Technology—Paraná, Cornélio Procópio, Brazil; 2 Big Data Brazil, São Paulo, SP, Brazil; 3 Institute of Computing, Federal University of Mato Grosso do Sul, Campo Grande, Brazil; 4 Department of Computing, São Paulo State University, Bauru, Brazil; 5 Institute of Computing, University of Campinas, Campinas, SP, Brazil; Leibniz Institute for Age Research, GERMANY

## Abstract

Nowadays, large datasets are common and demand faster and more effective pattern analysis techniques. However, methodologies to compare classifiers usually do not take into account the learning-time constraints required by applications. This work presents a methodology to compare classifiers with respect to their ability to learn from classification errors on a large learning set, within a given time limit. Faster techniques may acquire more training samples, but only when they are more effective will they achieve higher performance on unseen testing sets. We demonstrate this result using several techniques, multiple datasets, and typical learning-time limits required by applications.

## Introduction

Advances in digital technologies make large datasets commonly available, which demands faster and more effective pattern analysis techniques. However, methodologies to find the most suitable technique for a given application do not usually take into account the learning-time constraint required by the application. One may argue that parallel processing is possible in many situations and that machines are faster, but in practice datasets grow fast and opportunities for new applications continually emerge.

**Fig 1:**
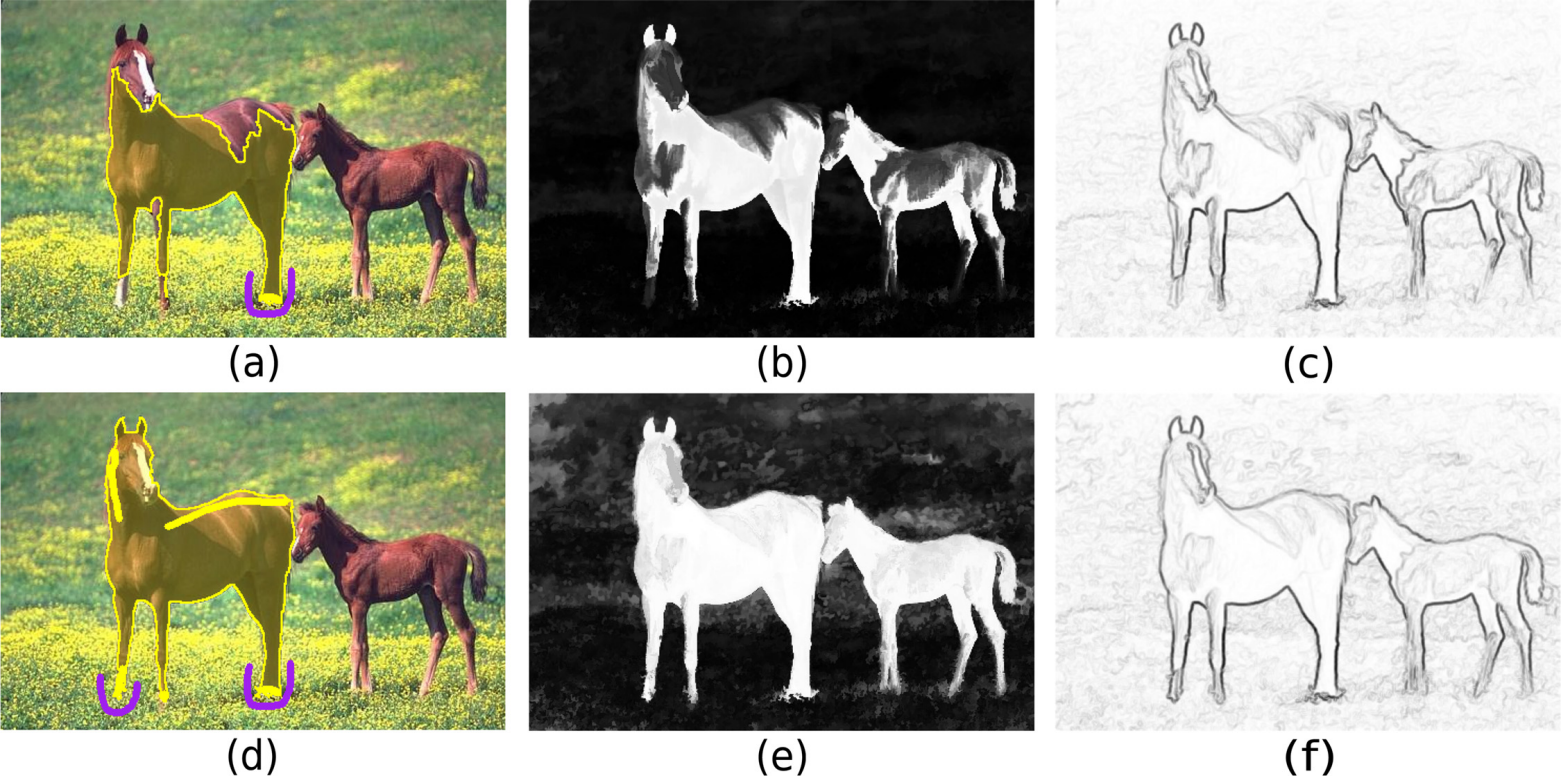
Example of interactive graph-based image segmentation. (a) The user draws labeled markers (a training set) inside and outside the object, and segmentation is based on optimum path competition from the markers in an image graph. (b) Image segmentation first relies on a pixel classifier, which is trained from the markers to create a fuzzy object map (the object should appear brighter than the background). (c) Second, the image is interpreted as a graph, whose arc weights should be lower on the border of the object than elsewhere. (d)-(f) The visual feedback from these results guides the user to the image location where more markers must be selected, improving fuzzy object map, arc weights, and so segmentation along a few interventions.

Consider a large database of face images obtained from many individuals through video cameras and all possible applications involving face recognition and verification. When a cell phone user uploads a video of her face to that database, such that a classifier can be trained to identify her and unlock the cell phone, the learning time for this application should not take more than a few seconds. In computer-assisted diagnosis of parasites [1], each microscopy slide may contain hundreds of thousands of image components to be classified either as impurity or as some type of parasite. Possible variations in the preparation of the slides, due to the choice of reagent brands or human operator, demand retraining and updating the classifier from time to time. The whole process should not take longer than a few minutes. Other applications, such as interactive segmentation of medical [2] and natural images [3], require real-time response to the user’s actions. Fig 1 illustrates an example based on the method described in [3]. The user draws labeled markers (a training set) inside and outside the object, and segmentation is based on optimum paths from the competing markers in an image graph (Fig 1a). These markers can easily contain tens of thousands of pixels, as modern digital cameras can take pictures with tens of millions of pixels. Image segmentation first relies on a pixel classifier, which is trained from the markers to create a fuzzy object map (Fig 1b). Second, the image is interpreted as a graph, whose pixels are the nodes and arcs between pixels are weighted based on intensity differences from the image and fuzzy object map (Fig 1c). For higher effectiveness, the object should appear brighter than the background in the fuzzy object map and arcs weights should be lower on the object’s border than elsewhere. The visual feedback from Fig 1a-1c guides the user to the image location where more markers must be selected, improving segmentation along a few interventions (Fig 1d-1f). Note that, the user should not have to wait longer than one second for a response after each intervention.

In general, applications in which a classifier must be trained upon user request (e.g., to answer a query performed by the user) should provide interactive response time. Even without user interaction, applications that require parameter optmization, using the accuracy of a classifier as criterion function, also require training and testing the classifier several times. As the dataset grow large, this becomes a problem. A good example of this occurs when learning the architecture of a convolutional neural network for feature extraction and classification—a hot topic nowadays. The response time does not need to be interactive, but processing time limitations may compromise the success of the optimization procedure by reducing the search space. Feature selection from large datasets may be used as a similar example. In the case of face recognition in mobile devices, this may be considered a future application that will become reality with the advances of cloud computing and communication networks. Currently, the mobile devices provide mechanisms for face recognition that are independent of the user—i.e., the design of the classifier does not consider the most informative samples to distinguish a particular user from other individuals with similar face characteristics. As a consequence of that, face recognition in mobile devices does not work properly. In order to have a robust face recognition system, it is desirable to learn the most informative samples from a large negative dataset, which could be stored and processed in a cloud system. During user enrollment, the important negative samples could be mined and together with the face samples from the user could train an user-specific classifier, which would be transmitted back to the mobile device, being the whole process performed in interactive time. Furthermore, for research purposes, where some techniques commonly take several minutes or hours to train a classifier, it is desirable to reduce the learning time to no longer than a few minutes. After all, we may need to repeat experiments hundreds of times in order to obtain statistically significant results and the increasing size of the datasets may also prevent us from train the classifier with all labeled samples.

Methodologies to compare pattern classification models usually fix features, training samples, test samples, and accuracy measures for all classifiers. This approach is adequate when evaluating the effectiveness of different techniques under the same conditions, but it contemplates neither learning-time constraints from the applications nor the fact that faster classifiers may be able to achieve higher performance on the same unseen testing set, given an allowance for a larger training set. For fairness with faster techniques and from a practical viewpoint, it is important to relax the constraint of a fixed training set for all classification models, provided that: (1) the training samples come from the same large learning set, and (2) all techniques must choose their own training samples and complete training within a pre-established learning-time limit, granted by the application.

In this work, we propose a methodology consistent with the above conditions. At first, a large dataset is randomly divided into learning and testing sets. The learning set should be large enough to contain representative samples from all classes. Given the learning-time limit allowed by a given application, it is usually not feasible to train a classifier using all learning samples. Therefore, the proposition is to start with a very small training set composed of randomly selected samples from the large learning set. This initial training set is the same for all classification models. Each classifier is then trained and subsequently evaluated on the learning set. By assembling a subset of randomly chosen misclassified samples, containing no more than the number of samples in the current training set, and incorporating it to the training set, we prepare the stage for a new learning cycle. This three-step procedure is then repeated until either (i) the learning-time limit from the application is reached or (ii) the number of errors becomes zero. In this way, faster techniques may acquire more error samples and complete the learning process with larger training sets. However, for their performance to be better on the unseen testing set, they must be more effective in learning from their own errors. Moreover, in order to achieve statistically significant results, this entire process, with distinct learning and test sets, has to be repeated several times.

We have evaluated our methodology on large datasets, using several techniques, and subject to the learning-time limits typically required by applications. We categorized these time limits into *very interactive* (less than 1s), *interactive* (from 1s to 5s), *nearly interactive* (from 5s to 1 minute), and *non-interactive* (above 1 minute). As this work aims at presenting a new methodology for the evaluation of classifiers and not to endorse any particular technique, we opted for standard implementations of each of the techniques used, since choosing optimized implementations might skew the results unfairly.

This work is organized as follows. We start by describing the background material and related works. Next, we introduce the new methodology. Then, we discuss the experiments and results. Finally, we present our conclusions.

## Background

Many works have presented pattern classification models based on discriminant analysis, nearest neighbors, neural networks, support vector machines and decision trees, among other techniques. In [4] the authors carry out a performance study of well-known classifiers, comparing the influence of the parameter configurations on the accuracy. They present a generic methodology to construct artificial datasets modeling the different characteristics of real data. Even though theoretical properties and settings may be used to justify new techniques, most of the literature compares the effectiveness of a proposed approach with respect to others for specific applications. Methodologies for that comparison usually divide a dataset into two parts, training and testing sets, where the first is used to project a classifier and the second to measure its errors [5]. This process must be repeated several times so that a sound conclusion on the statistics of its results can be reached.

Several aspects must be carefully considered for such methodologies to work. Firstly, call to mind that certain characteristics of the datasets (e.g., class imbalance) may require a specific sampling strategy [6]. Also, distinct sampling strategies to create training and testing sets can produce different estimates of performance [7]. The most popular ones are known as cross-validation, hold-out, leave-one-out, and bootstrap. Many articles adopt cross-validation techniques [8–11], despite their inherent trade-off regarding the number of folds and iterations. [9] showed that ten-fold cross-validation has a lower variance than leave-one-out and bootstrap. [10] and [11] hold running five separate iterations of two-fold cross-validation in order to reduce the correlation between the training sets.

On the other hand, one aspect rarely considered is that those methodologies are sensitive to the order of the samples in the dataset. [8] have evaluated the impact of the order of the samples in effectiveness, reproducibility, and generalization of the results. The authors showed that, due to distinct orders, a few iterations of cross-validation can severely affect the conclusions when comparing classifiers. [12] studied the consistency of statistical tests on individual datasets and recommended a corrected t-test [13] across ten iterations of ten-fold cross-validation as the least sensitive to the order of the samples. Other studies offered general guidelines for evaluation [14–17].

Different measures can also alter conclusions with respect to effectiveness. The literature offers many measures, such as: named accuracy [18–21], misclassification/error/hit rate [22], Mean Average Precision (MAP) [23], F-measure [24], H-measure [25], phi coefficient [26], Receiver Operating Characteristics (ROC) curve [27], Area Under the ROC Curve (AUC) [28–30], precision-recall curve [31, 32], while many other works [23, 25, 33–41] are devoted to optimizing the most popular measures. [14] discussed some measures in details and also pointed out how they differ, in order to define which one is the most suitable for a given experiment (application). Some works have also employed distinct measures according to each specific application domain [42].

The methodology presented in the next section can be used with any measure of effectiveness, since it is able to maximize effectiveness under a learning-time limit, as required by applications. To the best of our knowledge, our methodology is unique in the sense that it takes into account both the efficiency of the techniques in acquiring more knowledge (training samples) from the problem instance and their effectiveness in identifying the most representative training samples (errors in a learning set) to lead to a more accurate classifier. The methodologies mentioned above also aim at maximizing effectiveness, but they attempt this without regard for learning time. Given that each classifier selects its own training samples during the learning phase and the number of learning iterations is dependent on its efficiency, our methodology is robust with respect to the order of samples in the learning set. As we will see, the fact that all classifiers start with a small training set, obtained from randomly selected samples from the large learning set, considerably reduces the correlation between the initial training sets among multiple executions of the method.

## Methodology

In this section, we present the proposed evaluation methodology that considers efficacy and efficiency at the same time. In order to accomplish a fair comparison, the proposed methodology allows the classifiers to learn from their own errors, within a given time limit as required by the application.

A dataset 𝓩 is first randomly divided into a learning set 𝓩_2_ and a test set 𝓩_3_. Due to the learning-time limit of the given application, training with all learning samples is usually not possible. So, an initial training set 𝓩_1_ is created with a very small subset of randomly selected samples from 𝓩_2_, such that each class is represented by at least one sample.

After training each of the models with the same training set 𝓩_1_, they are evaluated on 𝓩_2_∖𝓩_1_. Next, we randomly select from the misclassified samples of each classifier a number of samples to be incorporated into (its own) 𝓩_1_ (this number is limited so as to, at most, double the size of the current training set). Retraining, evaluation, and misclassified sample selection is repeated until either the number of errors goes to zero or the learning-time limit 𝓣 of the application is reached. Within the learning-time limit 𝓣, each classifier has the opportunity to learn from its own classification errors on the learning set 𝓩_2_∖𝓩_1_, as the training set 𝓩_1_ increases.

This procedure works under the reasonable assumption that the most informative samples can be obtained from the errors on 𝓩_2_∖𝓩_1_. So, after each learning phase, an improvement in accuracy should also be expected on the unseen testing set 𝓩_3_. Algorithm 1 details this learning approach.

**Algorithm 1**: Learning Algorithm

**input**: A learning-time limit 𝒯, a learning set 𝓩_2_ and a function λ(*s*) that returns the correct label of any sample *s* ∈ 𝓩_2_.

**Output**: A supervised classifier.

**auxiliaries**: A training set 𝓩_1_ and a list 𝓜 of misclassified samples.

1 𝓩_1_ ← small random sampling from all classes in 𝓩_2_;

2 **repeat**

3   𝓜 ← ∅

4   Create a classifier instance 𝓘 from 𝓩_1_;

5   **for** each sample *t* ∈ 𝓩_2_∖𝓩_1_ **do**

6    Compute the label 𝓛(*t*) using 𝓘;

7    **if** 𝓛(*t*) ≠ *λ*(*t*) **then**

8     𝓜 ← 𝓜∪ {*t*}

9    **end**

10   **end**

11  𝓩_1_ ← 𝓩_1_∪(random subset of samples from 𝓜)

12 **until** (𝓜 = ∅ **or** learning time ≥ 𝓣);

## Experiments

In this section, we describe the overall experimental methodology, including datasets, effectiveness measure, classification models and the computational environment used. The experiments were carried out 100 times with randomly generated learning set 𝓩_2_ and test set 𝓩_3_. After starting off with an identical small training set containing at least one sample from each class, each individual classifier assembled its own training set 𝓩_1_ from 𝓩_2_. The resulting training sets ended up having different sizes, after the learning-time limit, according to the efficiency of each classifier. All the experiments reported here were performed on an off the shelf desktop computer featuring an Intel Core I5 processor and 4 GB of RAM. We used the standard C implementations of each classifier, as well as their own specific training strategy, with or without parameter optimization depending on the case, which is repeated during each learning iteration. It is impossible to establish the same parameter optimization method for all classifiers, because each classifier has its own mechanism. Some of them, such as OPF, does not require parameter optimization. Note that, since the methodology can be used to select the most suitable classifier for any given application, we are not targeting any application in particular. The experiments essentially demostrate the main characteristics of the methodology when comparing classifiers in different scenarios (datasets and time constraints).

### Dataset Description

For the experiments, we selected commonly available datasets of modest sizes with feature spaces of various dimensions.

- Cod-RNA Dataset [43]: comprised of 488,565 samples, 2 classes and 8 features.
- Connect-4 Dataset: obtained from the UCI Machine Learning Repository [44] containing 67,557 samples, 3 classes and 126 features.
- Covertype Dataset: also obtained from the UCI Machine Learning Repository [45]; it contains 581,012 samples, 7 classes and 54 features.
- IJCNN 2001 neural network competition [46]: consisting of 141,691 samples, 2 classes and 22 features.
- SensIT Vehicle (combined) [47]: made up of 98,528 samples, 3 classes and 100 features.

### Effectiveness Measure Description

It is important to highlight that the proposed methodology can be used with any effectiveness measure appropriate to the specific domain of application. The literature suggests several interesting effectiveness measures, as previously stated. In our experiments, we adopted the *F*_1_ score, which Jardine and van Rijsbergen [48] defined as the normalized, weighted harmonic mean of precision and recall:

$$\begin{array}{c} F_{1}=2*\frac{precision*recall}{precision+recall} \end{array}$$ (1)

### Learning-Time Constraints

For each dataset, we used four different learning-time limits, which were empirically chosen to simulate potential applications with different response times, so named: *very interactive*, *interactive*, *nearly interactive* and *non-interactive*. Table 1 presents the specified time limits for each type of application.

**Table 1:** Type of application and time limits.

Type of Application	Time Limit
Very Interactive	up to 1 sec
Interactive	1 sec to 5 sec
Nearly Interactive	5 sec to 60 sec
Non-Interactive	over 60 sec

For the sake of completion, the compared classifiers are briefly described in the following subsections. For more details, see [49, 50].

#### Support Vector Machines

Support Vector Machines (SVM) [51], a widely used classification model, is formulated as an optimization scheme that seeks to determine the hyperplane which best separates two classes (or one class from the others). Also, given non-linearly separable classes, it is possible to apply kernels that transform the data, improving separation between each class and the remaining ones. SVM’s main deficiency is that, depending on the size of the training set, too much computational time is needed for convergence to a solution. This lack of efficiency for large datasets may make SVM unfeasible in applications that require multiple retraining phases with interactive response times. Moreover, the assumption of class-separability in transformed space may not hold [52].

In our experiments, we used the latest version of the LibSVM package [49] with Gaussian mapping function (denoted as KSVM), optimization of the parameters 𝓒 and *γ* using 5-fold cross-validation within the training set and a grid search over exponentially growing sequences of 𝓒 and *γ* (𝓒 = 2^−5^,2^−3^, …, 2^15^ and *γ* = 2^−15^,2^−13^, …, 2^3^), as well as the linear version [53] of SVM (denoted as LSVM) and optimization of the parameter 𝓒 through 5-fold cross-validation. We also used a grid search over exponentially growing sequences of 𝓒.

#### *k*-nearest neighbors

The *k*-nearest neighbor (*k*-NN) algorithm is amongst the simplest and most largely used of all classification techniques. *k*-NN classifies a given sample by assigning it to the label most frequently present among its *k* nearest neighbors. For *k* = 1, a given sample is simply assigned to the class of its nearest neighbor and it corresponds to a first order Voronoi tesselation of the training data. *k*-NN takes into account *k* neighbors, so making the variance of the method less sensitive to noise and outliers. In this work, we estimated the value for *k* using a leave-one-out procedure over the training set (*k* = 1,3,5).

#### Optimum-Path Forest

The Optimum-Path Forest classifier (OPF) [50, 54] is a graph-based technique which models classification problems as optimum-path searches in graphs derived from an adjacency relation between samples in a given feature space (a complete relation, in this paper). The nodes are represented by the feature vectors and the edges connect pairs of them. Class representatives (prototypes) are chosen among the training samples in all classes and used to classify the remaining samples based on lengths of paths on the graph. This method has as advantage a very low computational training cost, given that it does not have to optimize parameters. Moreover, it can handle some overlap among classes.

In our experiments, we used LibOPF [50], which is a free library, implemented in the 𝓒 language, for the design of classifiers based on optimum-path forest. The distance between feature vectors was measured using log-euclidean distance.

## Results

In this section, we discuss the average results obtained by following the approach presented in the Methodology Section, with the experiments repeated 100 times with randomly selected learning and test sets. In the preprocessing step, we employed a feature standardization to avoid that attributes in larger numeric ranges dominate those in smaller ranges. We initialized each learning instance with 0.01% of 𝓩 for the initial training set, 𝓩_1_; 49.99%, for the learning set, 𝓩_2_∖𝓩_1_; and 50.0%, for the test set, 𝓩_3_. Given that each experiment was repeated 100 times with randomly selected learning and test sets, this procedure resembles a 50 × 2 Monte Carlo cross validation [55]. However, final training sets were constructed from this learning set by each classifier. Given that sample selection is based on a fixed increment of 0.01% of ∣𝓩_2_∣ (the learning set size) at each iteration, we can easily estimate the number of iterations, that each classifier required to complete the learning process (sample selection and training) within each given time limit, based on the final training set size. For instance, if the final training set contains 0.05% of the learning samples, then the classifier required 5 iterations.

**Fig 2:**
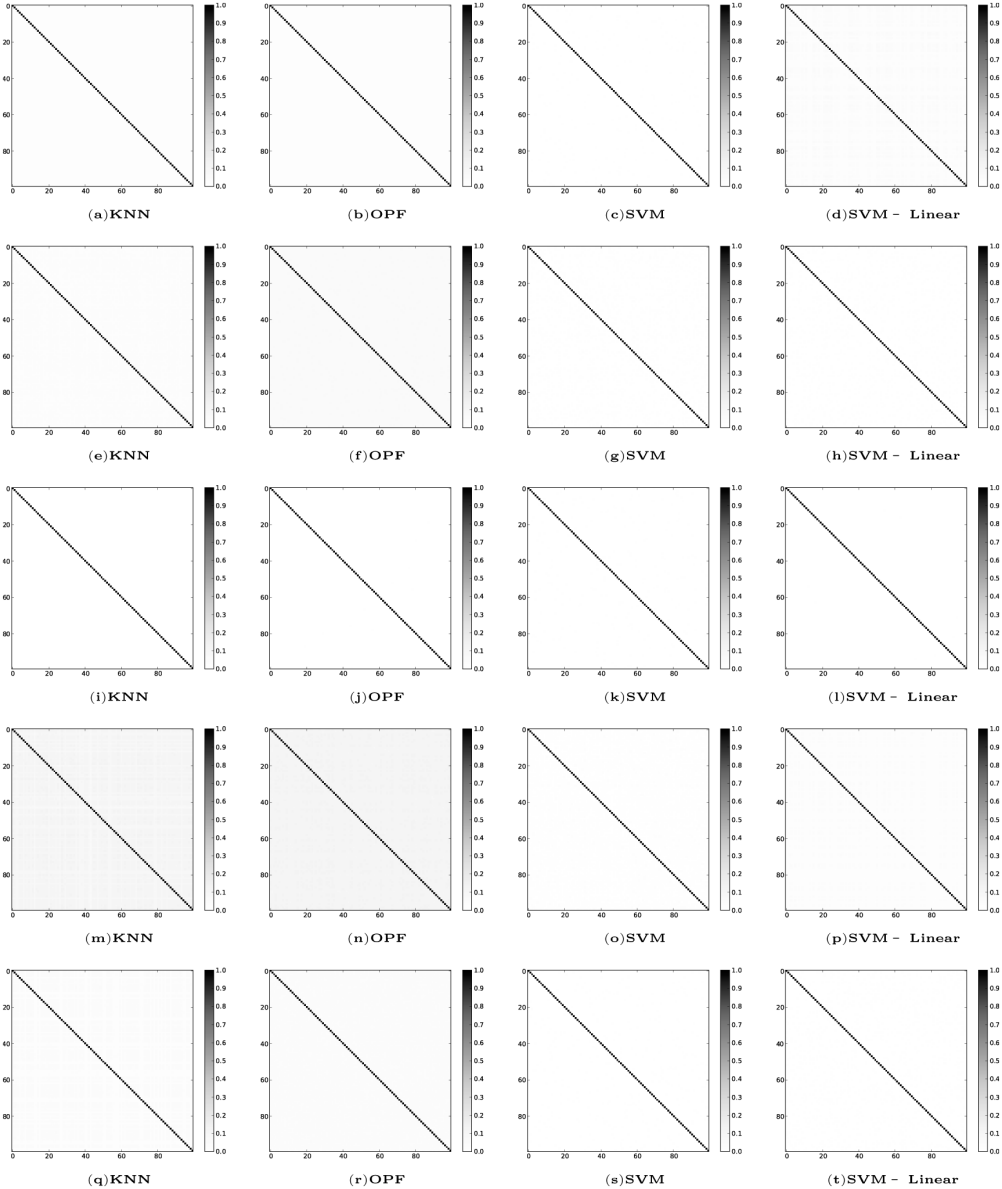
Correlation table between each pair of training sets, 𝓩_1_, after the learning process with learning constraint of 300 seconds. **Cod-RNA** (a—d). **Connect** (e—h). **Covertype** (i—l). **IJCNN** (m—p). **SensIT** (q—t).

Concerning the problem of overlapped training sets in cross-validation methodologies, as mentioned in the Background Section, we studied the correlation between each pair of training sets to evaluate the effectiveness of our methodology with respect to the choice of statistically different training sets (see Fig 2). The training sets present correlation below 0.2, being mostly much lower than that. This indicates that our methodology really measures the generalization ability of the classifiers for different training sets.

Tables 2–6 illustrate the effectiveness measure, namely the *F*_1_-score and the final training set size for each classification model, grouped by the learning-time constraint over different datasets.

From the experimental results for Cod-RNA dataset (Table 2), we see that both SVM strategies are able to obtain relatively good accuracy even with small training sets. One of the main issues with SVM is its non-scalability with respect to the number of training samples. Our methodology allowed these methods to select their most representative samples for a reduced training set.

**Table 2:** Cod-RNA dataset: Predictivity performance over 𝓩_3_ and final training set size, ∣𝓩_1_∣ as a percentual of ∣𝓩_2_∣, for each classification model and learning time constraint using the proposed selection.

Time constraint	Classifier	*F*_1_ *score*	∣𝓩_1_∣/∣𝓩_2_∣% (±variations)
1 sec	**KNN**	0.8190 ± 0.032	0.0393% ± 0.000
	**OPF**	0.8490 ± 0.013	0.0786% ± 0.000
	**LSVM**	**0.9342 ± 0.020**	0.0196% ± 0.000
	**KSVM**	**0.9279 ± 0.027**	0.0196% ± 0.000
5 sec	**KNN**	0.8673 ± 0.024	0.1902% ± 0.064
	**OPF**	0.8784 ± 0.006	0.3144% ± 0.000
	**LSVM**	**0.9272 ± 0.019**	0.1572% ± 0.000
	**KSVM**	**0.9346 ± 0.019**	0.0246% ± 0.009
20 sec	**KNN**	0.8958 ± 0.018	0.6288% ± 0.000
	**OPF**	0.8913 ± 0.005	0.6288% ± 0.000
	**LSVM**	**0.9338 ± 0.029**	0.6602% ± 0.137
	**KSVM**	**0.9402 ± 0.022**	0.0778% ± 0.006
60 sec	**KNN**	0.9100 ± 0.013	2.5151% ± 0.000
	**OPF**	**0.9131 ± 0.003**	2.5151% ± 0.000
	**LSVM**	**0.9401 ± 0.028**	2.4522% ± 0.274
	**KSVM**	**0.9492 ± 0.020**	0.0912% ± 0.029
300 sec	**KNN**	**0.9366 ± 0.006**	10.0605% ± 0.000
	**OPF**	**0.9333 ± 0.001**	10.0605% ± 0.000
	**LSVM**	**0.9198 ± 0.057**	9.1075% ± 1.867
	**KSVM**	**0.9495 ± 0.031**	0.2515% ± 0.077
1200 sec	**KNN**	**0.9640 ± 0.000**	18.9256% ± 0.546
	**OPF**	**0.9588 ± 0.005**	19.8643% ± 0.233
	**LSVM**	**0.9619 ± 0.007**	17.5507% ± 2.848
	**KSVM**	**0.9567 ± 0.028**	0.4205% ± 0.149

**Table 3:** Connect dataset: Predictivity performance over 𝓩_3_ and final training set size, ∣𝓩_1_∣ as a percentual of ∣𝓩_2_∣, for each classification model and learning time constraint using the proposed selection.

Time constraint	Classifier	*F*_1_ *score*	∣𝓩_1_∣/∣𝓩_2_∣% (± variations)
1 sec	**KNN**	**0.5443 ± 0.030**	0.2842% ± 0.000
	**OPF**	**0.5328 ± 0.017**	0.2842% ± 0.000
	**LSVM**	0.4364 ± 0.026	0.0178% ± 0.000
	**KSVM**	**0.5226 ± 0.000**	0.0178% ± 0.000
5 sec	**KNN**	**0.5704 ± 0.029**	1.1368% ± 0.000
	**OPF**	**0.5599 ± 0.008**	1.1368% ± 0.000
	**LSVM**	**0.5269 ± 0.027**	0.1229% ± 0.032
	**KSVM**	0.2614 ± 0.161	0.0355% ± 0.000
20 sec	**KNN**	**0.6046 ± 0.024**	4.5473% ± 0.000
	**OPF**	**0.5953 ± 0.005**	4.5473% ± 0.000
	**LSVM**	**0.6143 ± 0.032**	0.4860% ± 0.129
	**KSVM**	0.3277 ± 0.175	0.1435% ± 0.014
60 sec	**KNN**	**0.6263 ± 0.027**	9.0945% ± 0.000
	**OPF**	**0.6355 ± 0.003**	18.1891% ± 0.000
	**LSVM**	**0.6316 ± 0.019**	0.5741% ± 0.057
	**KSVM**	**0.5311 ± 0.107**	0.5684% ± 0.000
300 sec	**KNN**	**0.6828 ± 0.020**	36.3782% ± 0.000
	**OPF**	**0.6790 ± 0.002**	37.1242% ± 0.491
	**LSVM**	**0.6737 ± 0.012**	1.1368% ± 0.000
	**KSVM**	**0.6048 ± 0.087**	1.1368% ± 0.000
1200 sec	**KNN**	**0.7572 ± 0.018**	43.1991% ± 0.000
	**OPF**	**0.7772 ± 0.002**	43.1991% ± 0.000
	**LSVM**	0.5939 ± 0.037	6.5171% ± 2.187
	**KSVM**	0.6024 ± 0.044	2.6999% ± 0.000

**Table 4:** Covertype dataset: Predictivity performance over 𝓩_3_ and final training set size, ∣𝓩_1_∣ as a percentual of ∣𝓩_2_∣, for each classification model and learning time constraint using the proposed selection.

Time constraint	Classifier	*F*_1_ *score*	∣𝓩_1_∣/∣𝓩_2_∣% (± variations)
1 sec	**KNN**	**0.4842 ± 0.033**	0.0196% ± 0.000
	**OPF**	**0.5094 ± 0.024**	0.0196% ± 0.000
	**LSVM**	**0.5402 ± 0.031**	0.0196% ± 0.000
	**KSVM**	**0.5207 ± 0.070**	0.0196% ± 0.000
5 sec	**KNN**	**0.5200 ± 0.025**	0.0392% ± 0.000
	**OPF**	**0.5360 ± 0.020**	0.0392% ± 0.000
	**LSVM**	**0.5470 ± 0.032**	0.0196% ± 0.000
	**KSVM**	**0.5384 ± 0.058**	0.0196% ± 0.000
20 sec	**KNN**	**0.5680 ± 0.020**	0.1570% ± 0.000
	**OPF**	**0.5813 ± 0.012**	0.1570% ± 0.000
	**LSVM**	**0.5661 ± 0.030**	0.0502% ± 0.018
	**KSVM**	**0.5230 ± 0.063**	0.0196% ± 0.000
60 sec	**KNN**	0.6000 ± 0.018	0.3139% ± 0.000
	**OPF**	**0.6427 ± 0.015**	0.5776% ± 0.115
	**LSVM**	0.5969 ± 0.028	0.1319% ± 0.037
	**KSVM**	0.5226 ± 0.084	0.0392% ± 0.000
300 sec	**KNN**	**0.7156 ± 0.019**	2.5115% ± 0.000
	**OPF**	**0.7321 ± 0.003**	2.5115% ± 0.000
	**LSVM**	0.5959 ± 0.036	0.3186% ± 0.047
	**KSVM**	0.5780 ± 0.067	0.1570% ± 0.000
1200 sec	**KNN**	**0.8158 ± 0.025**	5.9734% ± 0.000
	**OPF**	**0.8374 ± 0.003**	5.9734% ± 0.000
	**LSVM**	0.6661 ± 0.019	23.8936% ± 0.000
	**KSVM**	0.6864 ± 0.016	0.3733% ± 0.000

**Table 5:** IJCNN dataset: Predictivity performance over 𝓩_3_ and final training set size, ∣𝓩_1_∣ as a percentual of ∣𝓩_2_∣, for each classification model and learning time constraint using the proposed selection.

Time constraint	Classifier	*F*_1_ *score*	∣𝓩_1_∣/∣𝓩_2_∣% (± variations)
1 sec	**KNN**	0.8800 ± 0.067	0.2936% ± 0.000
	**OPF**	0.8982 ± 0.039	0.2936% ± 0.000
	**LSVM**	0.8414 ± 0.044	0.0211% ± 0.007
	**KSVM**	**0.9497 ± 0.000**	0.0183% ± 0.000
5 sec	**KNN**	**0.9395 ± 0.024**	1.1744% ± 0.000
	**OPF**	**0.9433 ± 0.017**	1.1744% ± 0.000
	**LSVM**	**0.9257 ± 0.038**	0.2936% ± 0.000
	**KSVM**	**0.9319 ± 0.022**	0.0734% ± 0.000
20 sec	**KNN**	**0.9661 ± 0.013**	4.6975% ± 0.000
	**OPF**	**0.9688 ± 0.006**	4.6975% ± 0.000
	**LSVM**	**0.8949 ± 0.076**	1.1744% ± 0.000
	**KSVM**	**0.9528 ± 0.024**	0.2936% ± 0.000
60 sec	**KNN**	**0.9809 ± 0.009**	11.1440% ± 1.406
	**OPF**	**0.9852 ± 0.001**	12.3998% ± 0.497
	**LSVM**	0.8804 ± 0.092	2.3723% ± 0.234
	**KSVM**	0.9619 ± 0.016	0.5872% ± 0.000
300 sec	**KNN**	**0.9894 ± 0.001**	13.6423% ± 1.607
	**OPF**	0.9883 ± 0.000	13.4193% ± 0.371
	**LSVM**	0.8571 ± 0.098	9.3951% ± 0.000
	**KSVM**	0.9769 ± 0.008	1.1744% ± 0.000
1200 sec	**KNN**	**0.9896 ± 0.001**	13.7359% ± 1.579
	**OPF**	0.9883 ± 0.000	13.4822% ± 0.404
	**LSVM**	0.9571 ± 0.001	22.8248% ± 2.280
	**KSVM**	0.9756 ± 0.010	2.3488% ± 0.000

**Table 6:** SensIT dataset: Predictivity performance over 𝓩_3_ and final training set size, ∣𝓩_1_∣ as a percentual of ∣𝓩_2_∣, for each classification model and learning time constraint using the proposed selection.

Time constraint	Classifier	*F*_1_ *score*	∣𝓩_1_∣/∣𝓩_2_∣% (± variations)
1 sec	**KNN**	**0.4387 ± 0.035**	0.1462% ± 0.000
	**OPF**	**0.4367 ± 0.025**	0.1462% ± 0.000
	**LSVM**	**0.4124 ± 0.055**	0.0183% ± 0.000
	**KSVM**	**0.3667 ± 0.089**	0.0183% ± 0.000
5 sec	**KNN**	**0.4701 ± 0.026**	0.5846% ± 0.000
	**OPF**	**0.4621 ± 0.015**	0.5846% ± 0.000
	**LSVM**	**0.4261 ± 0.036**	0.0731% ± 0.000
	**KSVM**	0.3126 ± 0.111	0.0353% ± 0.005
20 sec	**KNN**	**0.4942 ± 0.020**	2.3384% ± 0.000
	**OPF**	**0.4793 ± 0.008**	2.3384% ± 0.000
	**LSVM**	**0.4887 ± 0.021**	0.5320% ± 0.112
	**KSVM**	**0.4285 ± 0.085**	0.1462% ± 0.000
60 sec	**KNN**	**0.5076 ± 0.020**	7.7636% ± 2.215
	**OPF**	**0.4903 ± 0.005**	9.3537% ± 0.000
	**LSVM**	**0.4945 ± 0.019**	0.5846% ± 0.000
	**KSVM**	**0.4475 ± 0.077**	0.2923% ± 0.000
300 sec	**KNN**	**0.5089 ± 0.023**	18.7074% ± 0.000
	**OPF**	**0.4966 ± 0.003**	37.4147% ± 0.000
	**LSVM**	**0.5332 ± 0.018**	1.1692% ± 0.000
	**KSVM**	**0.4729 ± 0.072**	1.1692% ± 0.000
1200 sec	**KNN**	**0.5169 ± 0.022**	63.4233% ± 2.274
	**OPF**	**0.5100 ± 0.002**	71.4498% ± 0.233
	**LSVM**	**0.5361 ± 0.021**	2.3384% ± 0.000
	**KSVM**	**0.5200 ± 0.050**	2.3384% ± 0.000

In Table 3, we can see that faster techniques, such as OPF and *k*-NN, can acquire more samples within the time constraint as well as achieve higher mean accuracy. However, *k*-NN usually presents higher variance, being more sensitive to noise. Differently, OPF presents a more stable performance (Tables 3–6), in general, especially in multi-class problems.

Some techniques can learn faster than others, building larger training sets. However, the ability of the technique in selecting the most informative samples is more important than its speed. This makes an interesting point with respect to the proposed methodology. It is fair to all techniques in the sense that each one has the chance to mine the most informative samples for training. Note that, Tables 2–6, show the final training set size of each technique and the best technique is not always the one with largest training set. Indeed, faster techniques obtained their maximal predictive performance only when they could effectively learn from their errors.

To provide a statistical analysis of the results, we performed a Friedman test [56] for each pair of dataset and learning time constraint. Demšar [57] states that the Friedman test provides reliable conclusions when the assumptions (normal distributions and sphericity) of the traditional multiple hypotheses testing ANOVA are violated.

**Fig 3:**
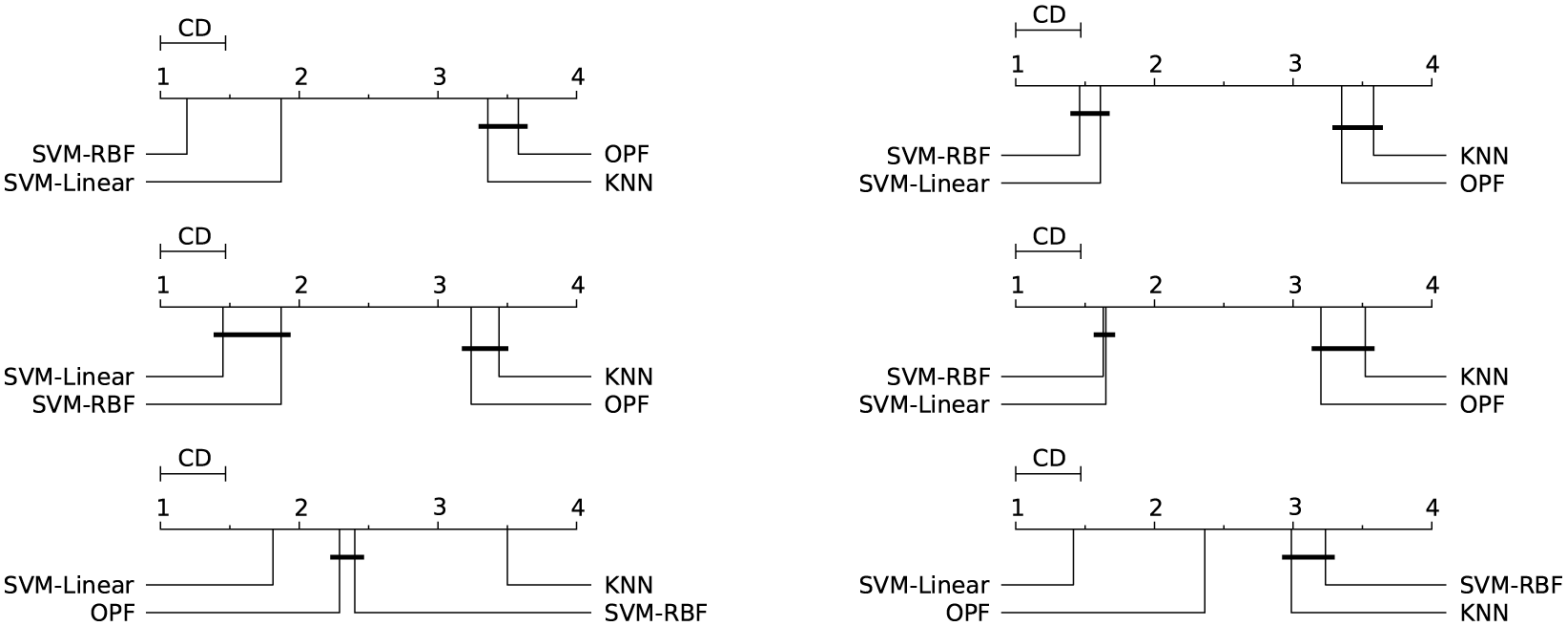
Cod-RNA. Comparison of all classifiers against each other with the Nemenyi test and learning time constraint equals to 1, 5, 20, 60, 300, and 1200 seconds. Groups of classifiers that are not significantly different (at p = 0.05) are connected.

**Fig 4:**
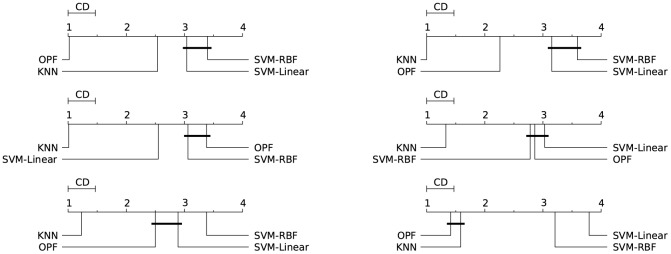
Connect-4. Comparison of all classifiers against each other with the Nemenyi test and learning time constraint equals to 1, 5, 20, 60, 300, and 1200 seconds. Groups of classifiers that are not significantly different (at p = 0.05) are connected.

**Fig 5:**
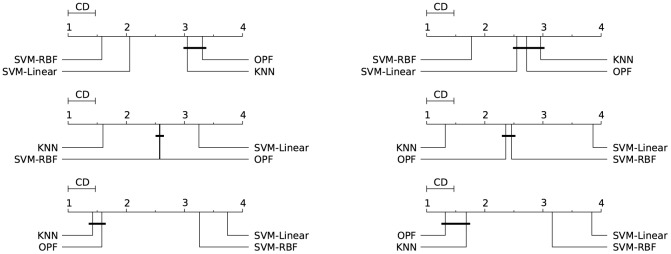
Covertype. Comparison of all classifiers against each other with the Nemenyi test and learning time constraint equals to 1, 5, 20, 60, 300, and 1200 seconds. Groups of classifiers that are not significantly different (at p = 0.05) are connected.

**Fig 6:**
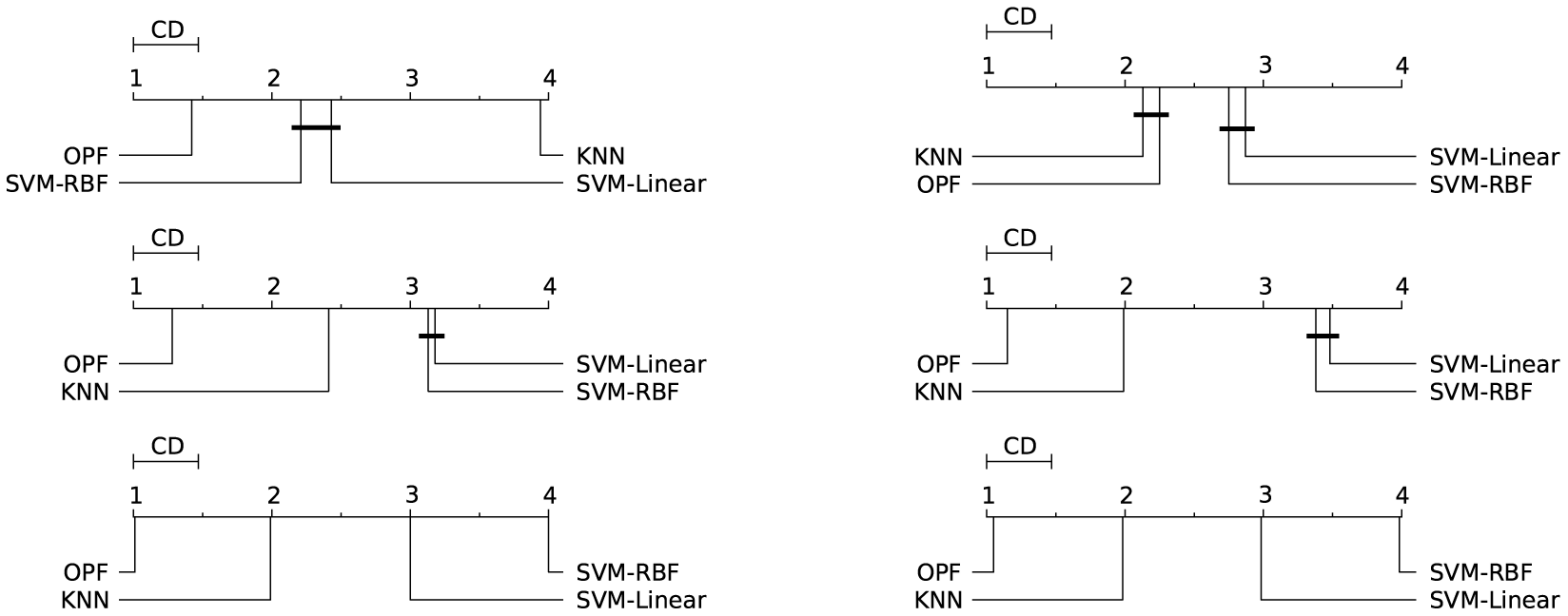
IJCNN 2001. Comparison of all classifiers against each other with the Nemenyi test and learning time constraint equals to 1, 5, 20, 60, 300, and 1200 seconds. Groups of classifiers that are not significantly different (at p = 0.05) are connected.

**Fig 7:**
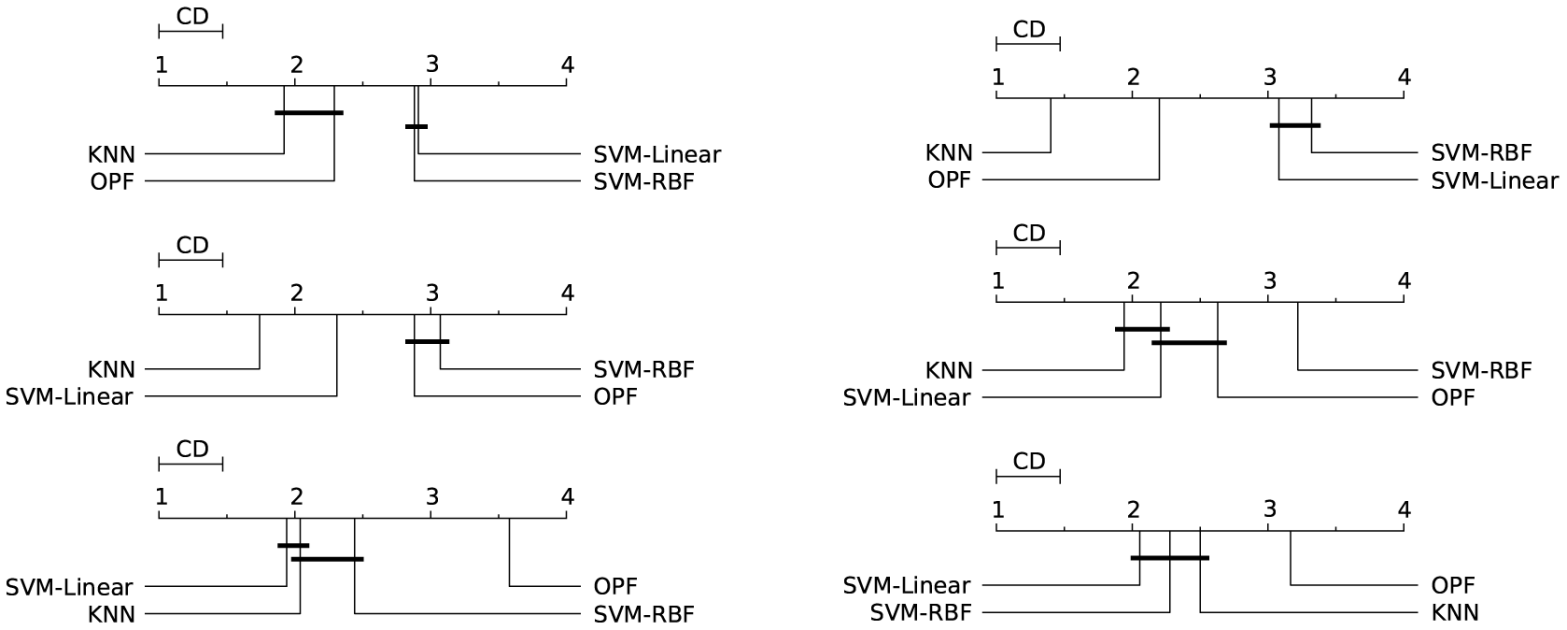
SensIT Vehicle (combined). Comparison of all classifiers against each other with the Nemenyi test and learning time constraint equals to 1, 5, 20, 60, 300, and 1200 seconds. Groups of classifiers that are not significantly different (at p = 0.05) are connected.

Figs 3–7 illustrate a graphical representation of the post-hoc Nemenyi test [58], since we rejected the null hypotheses that all the classifiers are equivalent. Note that 1 represents the best technique, and while 4 stands for the worst one. Groups of classifiers that are not significantly different (at *p* = 0.05) are connected by using a calculated critical distance (CD) equals to 0.4690.

It is worth noting the importance of the statistical test, since the mean and standard deviation (see Tables 2–6) in some cases are not sufficient to indicate the best classifier. The results presented by both tests (Nemenyi test and mean-standard deviation), in general, are equivalents. The statistical test in the CodRNA dataset (Fig 3) shows that both SVMs have significantly better performance when compared to other classifiers. According to the mean and standard deviation (Table 2), both SVMs are equivalent. However, the statistical tests show evidence that they are not.

Such divergences can also be observed with the other datasets. They occur due to the fact that the standard deviation values are relatively high compared to the difference in performance of the classifiers. Similarly to the results expressed by the mean and standard deviation (Tables 3–6), the statistical test also reveals that *k*-NN and OPF, in general, present a better performance for Connect, Covertype, IJCNN and SensIT Vehicle datasets (Figs 4–7). However, the Nemenyi test indicates statistically significant differences between them, unlike the mean-standard deviation test. It is important to clarify that in some cases, for example SensIT Vehicle dataset with learning-time constraint 300, we cannot reach any conclusion regarding the relative performances of LSVM, *k*-NN and OPF.

Sample selection methods do not account for time constraints. Methods based on clustering and statistical information learned from the data are usually time costly for large learning sets, which would make it very difficult to select and train a classifier within lower time limits. The simplest approach is random sample selection from each class. Even in this case, one has to estimate the maximum number of samples that a given model can use to train the classifier in a single iteration and within the given time limit. First, for some models, such as SVM, the training time also depends on the selected samples. Anyway, ignoring that, we have estimated that number for each classification model and compared to the proposed sample selection approach based on classification errors. Tables 7–11 present the corresponding results using a single learning iteration with the maximum number of randomly selected samples.

**Table 7:** Cod-RNA dataset: Predictivity performance over 𝓩_3_ and final training set size, ∣𝓩_1_∣ as a percentual of ∣𝓩_2_∣, for each classification model and learning time constraint using random selection.

Time constraint	Classifier	*F*_1_ *score*	∣𝓩_1_∣/∣𝓩_2_∣% (± variations)
1 sec	**KNN**	0.7863 ± 0.024	0.4300% ± 0.068
	**OPF**	0.8107 ± 0.032	0.9003% ± 0.085
	**LSVM**	0.8983 ± 0.041	0.4303% ± 0.220
	**KSVM**	0.8499 ± 0.118	0.1498% ± 0.066
5 sec	**KNN**	0.8432 ± 0.014	3.0301% ± 0.116
	**OPF**	0.8531 ± 0.019	4.6503% ± 0.195
	**LSVM**	0.9329 ± 0.006	2.6303% ± 0.116
	**KSVM**	0.9387 ± 0.005	1.3901% ± 0.166
20 sec	**KNN**	0.8728 ± 0.011	11.3300% ± 0.221
	**OPF**	0.8781 ± 0.017	17.2904% ± 0.152
	**LSVM**	0.9377 ± 0.002	9.9906% ± 0.100
	**KSVM**	0.9449 ± 0.001	4.2096% ± 0.099
60 sec	**KNN**	0.8818 ± 0.007	35.2902% ± 0.166
	**OPF**	0.8940 ± 0.007	40.2902% ± 0.152
	**LSVM**	0.9385 ± 0.005	24.9295% ± 0.095
	**KSVM**	0.9490 ± 0.001	8.7100% ± 0.152
300 sec	**KNN**	0.8998 ± 0.000	100.0000% ± 0.000
	**OPF**	0.8955 ± 0.000	100.0000% ± 0.000
	**LSVM**	0.9357 ± 0.008	70.2701% ± 0.274
	**KSVM**	0.9518 ± 0.008	19.2902% ± 0.251
1200 sec	**KNN**	0.8998 ± 0.000	100.0000% ± 0.000
	**OPF**	0.8955 ± 0.000	100.0000% ± 0.000
	**LSVM**	0.9397 ± 0.000	100.0000% ± 0.000
	**KSVM**	0.9533 ± 0.004	44.2700% ± 0.275

**Table 8:** Connect dataset: Predictivity performance over 𝓩_3_ and final training set size, ∣𝓩_1_∣ as a percentual of ∣𝓩_2_∣, for each classification model and learning time constraint using random selection.

Time constraint	Classifier	*F*_1_ *score*	∣𝓩_1_∣/∣𝓩_2_∣% (± variations)
1 sec	**KNN**	0.5132 ± 0.274	2.0799% ± 0.093
	**OPF**	0.5157 ± 0.028	2.8840% ± 0.067
	**LSVM**	0.5608 ± 0.028	0.1504% ± 0.066
	**KSVM**	0.5065 ± 0.024	0.0975% ± 0.021
5 sec	**KNN**	0.5593 ± 0.019	4.6868% ± 0.302
	**OPF**	0.5469 ± 0.014	6.7665% ± 0.211
	**LSVM**	0.6167 ± 0.012	0.4902% ± 0.074
	**KSVM**	0.3749 ± 0.001	0.3296% ± 0.289
20 sec	**KNN**	0.6493 ± 0.006	9.0972% ± 0.302
	**OPF**	0.5871 ± 0.007	14.0972% ± 0.441
	**LSVM**	0.6799 ± 0.001	1.1797% ± 0.051
	**KSVM**	0.4246 ± 0.001	1.1501% ± 0.553
60 sec	**KNN**	0.6697 ± 0.002	18.6447% ± 0.307
	**OPF**	0.6234 ± 0.004	25.7391% ± 0.203
	**LSVM**	0.6867 ± 0.002	2.4295% ± 0.067
	**KSVM**	0.6024 ± 0.006	2.2500% ± 0.113
300 sec	**KNN**	0.7321 ± 0.001	80.9282% ± 0.655
	**OPF**	0.6927 ± 0.000	100.0000% ± 0.000
	**LSVM**	0.6992 ± 0.005	9.5821% ± 0.103
	**KSVM**	0.6189 ± 0.006	5.9501% ± 0.110
1200 sec	**KNN**	0.7425 ± 0.001	100.0000% ± 0.000
	**OPF**	0.6927 ± 0.000	100.0000% ± 0.000
	**LSVM**	0.7106 ± 0.001	25.3101% ± 0.232
	**KSVM**	0.6756 ± 0.008	11.1401% ± 0.303

**Table 9:** Covertype dataset: Predictivity performance over 𝓩_3_ and final training set size, ∣𝓩_1_∣ as a percentual of ∣𝓩_2_∣, for each classification model and learning time constraint using random selection.

Time constraint	Classifier	*F*_1_ *score*	∣𝓩_1_∣/∣𝓩_2_∣% (± variations)
1 sec	**KNN**	0.5057 ± 0.059	0.6516% ± 0.084
	**OPF**	0.4906 ± 0.014	0.6523% ± 0.303
	**LSVM**	0.5306 ± 0.070	0.0699% ± 0.147
	**KSVM**	0.5378 ± 0.037	0.0596% ± 0.211
5 sec	**KNN**	0.5145 ± 0.009	1.3241% ± 0.018
	**OPF**	0.5029 ± 0.025	1.5392% ± 0.012
	**LSVM**	0.5461 ± 0.065	0.1196% ± 0.371
	**KSVM**	0.5392 ± 0.059	0.1021% ± 0.514
20 sec	**KNN**	0.5767 ± 0.006	2.2071% ± 0.053
	**OPF**	0.5651 ± 0.051	3.1570% ± 0.219
	**LSVM**	0.6183 ± 0.020	0.3802% ± 0.068
	**KSVM**	0.5304 ± 0.053	0.3124% ± 0.145
60 sec	**KNN**	0.6328 ± 0.015	5.6238% ± 0.166
	**OPF**	0.6192 ± 0.065	5.5776% ± 0.067
	**LSVM**	0.6414 ± 0.008	0.7019% ± 0.211
	**KSVM**	0.6504 ± 0.046	0.6491% ± 0.251
300 sec	**KNN**	0.6821 ± 0.029	9.5115% ± 0.476
	**OPF**	0.6949 ± 0.007	10.5115% ± 0.052
	**LSVM**	0.6478 ± 0.007	2.1687% ± 0.303
	**KSVM**	0.6674 ± 0.018	1.1007% ± 0.303
1200 sec	**KNN**	0.7454 ± 0.035	19.9734% ± 0.023
	**OPF**	0.7587 ± 0.097	20.9734% ± 0.226
	**LSVM**	0.6944 ± 0.003	5.7300% ± 0.116
	**KSVM**	0.6776 ± 0.016	3.8273% ± 0.032

**Table 10:** IJCNN dataset: Predictivity performance over 𝓩_3_ and final training set size, ∣𝓩_1_∣ as a percentual of ∣𝓩_2_∣, for each classification model and learning time constraint using random selection.

Time constraint	Classifier	*F*_1_ *score*	∣𝓩_1_∣/∣𝓩_2_∣% (± variations)
1 sec	**KNN**	0.8621 ± 0.012	0.9502% ± 0.110
	**OPF**	0.8637 ± 0.006	0.9582% ± 0.120
	**LSVM**	0.8888 ± 0.016	0.7301% ± 0.257
	**KSVM**	0.8647 ± 0.017	0.0320% ± 0.134
5 sec	**KNN**	0.9070 ± 0.005	4.6509% ± 0.303
	**OPF**	0.9039 ± 0.006	5.4511% ± 0.196
	**LSVM**	0.8654 ± 0.100	2.9706% ± 0.221
	**KSVM**	0.9244 ± 0.018	1.7504% ± 0.301
20 sec	**KNN**	0.9340 ± 0.004	15.2490% ± 0.346
	**OPF**	0.9313 ± 0.005	15.4491% ± 0.127
	**LSVM**	0.8944 ± 0.012	9.9100% ± 0.250
	**KSVM**	0.9558 ± 0.003	6.4513% ± 0.269
60 sec	**KNN**	0.9527 ± 0.002	36.0492% ± 0.508
	**OPF**	0.9494 ± 0.003	36.4493% ± 0.158
	**LSVM**	0.8988 ± 0.040	24.4509% ± 0.221
	**KSVM**	0.9612 ± 0.004	10.4485% ± 0.377
300 sec	**KNN**	0.9655 ± 0.000	100.0000% ± 0.000
	**OPF**	0.9624 ± 0.000	100.0000% ± 0.000
	**LSVM**	0.9110 ± 0.004	72.2685% ± 0.275
	**KSVM**	0.9724 ± 0.001	25.4511% ± 0.504
1200 sec	**KNN**	0.9655 ± 0.000	100.0000% ± 0.000
	**OPF**	0.9624 ± 0.000	100.0000% ± 0.000
	**LSVM**	0.9168 ± 0.000	100.0000% ± 0.000
	**KSVM**	0.9782 ± 0.001	65.4511% ± 0.238

**Table 11:** SensIT dataset: Predictivity performance over 𝓩_3_ and final training set size, ∣𝓩_1_∣ as a percentual of ∣𝓩_2_∣, for each classification model and learning time constraint using random selection.

Time constraint	Classifier	*F*_1_ *score*	∣𝓩_1_∣/∣𝓩_2_∣% (± variations)
1 sec	**KNN**	0.4100 ± 0.038	0.5498% ± 0.083
	**OPF**	0.4439 ± 0.013	1.5500% ± 0.031
	**LSVM**	0.3803 ± 0.087	0.1497% ± 0.054
	**KSVM**	0.4261 ± 0.040	0.1501% ± 0.303
5 sec	**KNN**	0.4662 ± 0.007	3.0501% ± 0.047
	**OPF**	0.4668 ± 0.006	4.0500% ± 0.051
	**LSVM**	0.4029 ± 0.085	0.2898% ± 0.074
	**KSVM**	0.4372 ± 0.029	0.2335% ± 0.008
20 sec	**KNN**	0.4875 ± 0.005	7.5500% ± 0.069
	**OPF**	0.4823 ± 0.006	8.5499% ± 0.054
	**LSVM**	0.4931 ± 0.011	0.8696% ± 0.116
	**KSVM**	0.4636 ± 0.010	0.7202% ± 0.275
60 sec	**KNN**	0.5031 ± 0.005	14.5501% ± 0.072
	**OPF**	0.4849 ± 0.034	16.0002% ± 0.110
	**LSVM**	0.5234 ± 0.004	1.6305% ± 0.067
	**KSVM**	0.4788 ± 0.004	1.4699% ± 0.133
300 sec	**KNN**	0.5207 ± 0.003	40.4501% ± 0.021
	**OPF**	0.5072 ± 0.067	44.9990% ± 0.013
	**LSVM**	0.5409 ± 0.003	5.6696% ± 0.149
	**KSVM**	0.4905 ± 0.006	3.5498% ± 0.075
1200 sec	**KNN**	0.5301 ± 0.001	90.4501% ± 0.528
	**OPF**	0.5185 ± 0.000	100.0000% ± 0.000
	**LSVM**	0.5473 ± 0.002	16.3701% ± 0.221
	**KSVM**	0.5034 ± 0.004	8.4500% ± 0.717

Comparing the results achieved by the proposed method (Tables 2–6) with the ones by the randomized method (Tables 7–11), one can observe that in general, the proposed methodology is capable to select the most representative samples for the training set, holding higher accuracy results (see Tables 2 and 7 with time constraint equal to 1 sec for all classifiers). Even in some cases, when it was possible to train with the entire dataset (for instance, see Table 7 with time constraint equal to 300 sec for *k*-NN and OPF, as well as with time constraint equal to 1200 sec for *k*-NN, OPF and LSVM), it seems that some randomly selected training samples impaired the performance of the classifier, while our methodology is capable to avoid them in the training set (see Table 2 with the same time constraints and classifiers). Note also that the proposed methodology can output considerably smaller training sets, which matters in some approaches, such as the OPF and *k*-NN classifiers, to speed up classification of large test sets.

**Fig 8:**
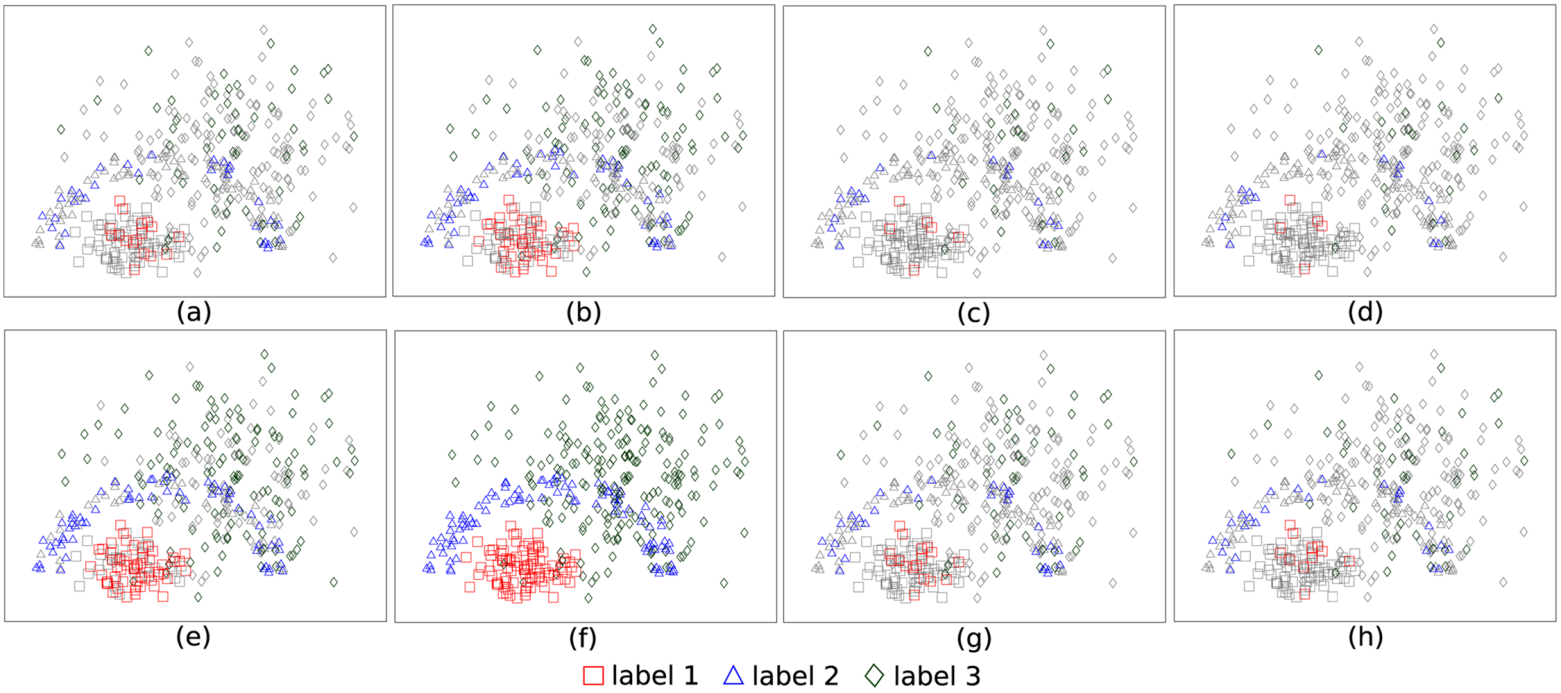
Randomly selected samples in the final training set when using the Cone-Torus dataset. (a) *k*-NN and 1 sec. (b) OPF and 1 sec. (c) LSVM and 1 sec. (d) KSVM and 1 sec. (e) *k*-NN and 1.5 sec. (f) OPF and 1.5 sec. (g) LSVM and 1.5 sec. (h) KSVM and 1.5 sec.

The comparison of methods using randomized sample selection is not suitable, because these samples capture the geometry of the classes. Besides, as they increase in number, all classification models become equivalent. Fig 8 shows randomly selected samples by each classification model within a given time constraint (1 and 1.5 seconds) using the Cone-Torus dataset [59]. Samples that were not selected are highlighted in gray. One can observe that OPF reached greater effectiveness and efficiency, selecting 100% of the learning samples with only 1.5 seconds and presenting F-score measure equal to 1 (Table 12). It is noteworthy that faster techniques (with a larger training set) do not always achieve higher accuracy. It relies on the effective learning from their errors. For instance, LSVM did not achieved better performance, even being faster than KSVM.

**Table 12:** Cone-Torus dataset: Predictivity performance over 𝓩_3_ and final training set size, ∣𝓩_1_∣ as a percentual of ∣𝓩_2_∣, for each classification model using random selection and learning time constraint equals to 1 and 1.5 seconds.

Time constraint	Classifier	*F*_1_ *score*	∣𝓩_1_∣/∣𝓩_2_∣% (± variations)
1 sec	**KNN**	0.9303	25%
	**OPF**	1.0000	50%
	**LSVM**	0.6092	12%
	**KSVM**	0.7875	10%
1.5 sec	**KNN**	0.9389	60%
	**OPF**	1.0000	100%
	**LSVM**	0.6485	25%
	**KSVM**	0.8147	20%

**Fig 9:**
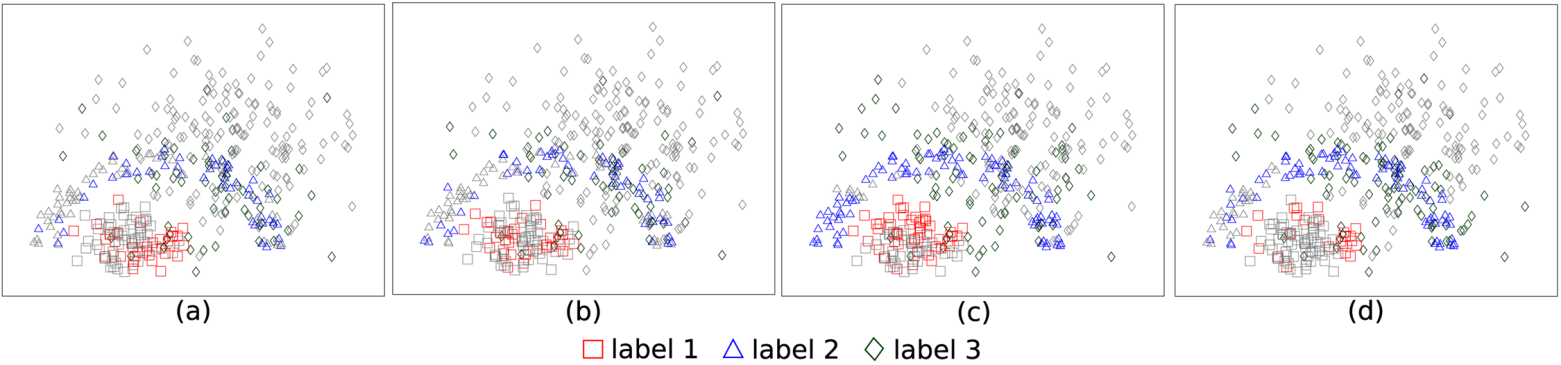
Selected samples by each classification model in the final training set, when using the Cone-Torus dataset and time limit of 1s. (a) *k*-NN. (b) OPF. (c) LSVM. (d) KSVM.

Each classification model defines decision boundaries (regions) in a different way in the feature space. By selecting classification errors as training samples, the learning process converges faster to the corresponding decision boundaries. The errors tend to be samples close to the decision boundaries rather than outliers, as long as outliers are minority. If this is not the case, outlier removal should be applied before the learning process. In order to better clarify this issue, we have added Fig 9 with samples not selected from the learning set in gray and samples selected by the classifiers to the training set in color. Fig 9 shows the selected samples for 1 second of time limit using the 2D Cone-Torus dataset.

In order to analyze the performance of each classifier using the entire learning set, Table 13 shows the accuracy and the time required for training each dataset. All classifiers presented similar accuracies. However, *k*-NN and OPF were more efficient. KSVM and LSVM require significantly more time for training.

**Table 13:** Predictivity performance over 𝓩_3_ and required time for each classification model and dataset using all learning samples, ∣𝓩_2_∣.

Datasets	Classifier	*F*_1_ *score*	Time (sec)
Cod-RNA	**KNN**	**0.8998**	**165.01**
	**OPF**	**0.8955**	**148.92**
	**LSVM**	**0.9397**	426.92
	**KSVM**	**0.9598**	1555.19
Connect	**KNN**	**0.7425**	**369.66**
	**OPF**	**0.6927**	**233.12**
	**LSVM**	**0.7845**	4698.15
	**KSVM**	**0.7969**	10651.79
Covertype	**KNN**	**0.8358**	**6016.87**
	**OPF**	**0.8401**	**5832.24**
	**LSVM**	**0.8662**	21813.39
	**KSVM**	**0.8773**	32149.23
IJCNN	**KNN**	**0.9655**	**168.62**
	**OPF**	**0.9624**	**165.04**
	**LSVM**	**0.9168**	425.71
	**KSVM**	**0.9802**	1724.40
SensIT	**KNN**	**0.5367**	**1403.79**
	**OPF**	**0.5181**	**689.14**
	**LSVM**	**0.5899**	7462.38
	**KSVM**	**0.6014**	15521.22

## Conclusion

We presented a methodology to compare multiple classifiers under a learning-time constraint, which is useful to select the best classifier for a given application. In this paper, the applications were represented by different datasets with unbalancing of classes, distinct number of classes and feature space dimensions. The proposed methodology allows each classifier to select its most representative samples from a learning set during the training phase. The experiments allowed us to reach several conclusions.

Although it was not possible to assert which is the most effective classification model under a given time constraint, due to the variability of results on each application domain, experiments obtained using the proposed methodology allowed us to arrive at some relevant observations.

Larger training sets do not necessarily lead to higher predictive performance on unseen test sets, which indicates the effectiveness of some classifiers in learning from their own errors.

The methodology is able to produce statistically independent training sets as observed by the low correlations between each pair of training set obtained for a given dataset-classifier pair, following 100 executions. This demonstrates the advantage of our approach with respect to the regular cross-validation procedure, largely used in related works.

It is also very common in the literature for the presentation of experimental results to rely solely on the mean and standard deviation of accuracy values. The statistical test shows that this approach is not always reliable, due to the relative variations of the standard deviation.
